# Cases and Observations, Illustrative of Renal Disease, Accompanied with the Secretion of Albuminous Urine

**Published:** 1839-07

**Authors:** 


### ART. VI.

1. *Cases and Observations, illustrative of Renal Disease, accompanied with the Secretion of Albuminous Urine.* By Dr. BRIGHT. (Guy's Hospital Reports, No. II., 1836.)

2. *De l'Albuminurie ou Hydropisie causée par Maladie des Reins, &c.* Par le Dr. MARTIN SOLON, Médecin de l'Hôpital Beaujon, &c. *Paris*, 1838. 8vo, pp. 480. *Avec planches coloriées.*
*On Albuminuria, or Dropsy, caused by diseased Kidney.* By M. SOLON, M.D.

3. *Traité des Maladies des Reins et des Altérations de la Secrétion Urinaire, &c., avec un Atlas in Folio.* Par P. RAYER, Médecin de l'Hôpital de la Charité, &c. Tome 1er, pp. 625. *Paris*, 1839.
*Treatise on Diseases of the Kidneys and the Morbid States of the Urinary Secretion, &c.* By P. RAYER, M.D., &c.

4. *On Granular Degeneration of the Kidneys, and its connexion with Dropsy, Inflammations, and other Diseases.* By ROBERT CHRISTISON, M.D. F.R.S.E., &c. *Edinburgh*, 1839. 8vo, pp. 288.

5. *Observations on Abdominal Tumours and Intumescence; illustrated by cases of Renal Disease.* By R. BRIGHT, M.D. F.R.S., &c. (Guy's Hospital Reports, No. VIII., April, 1839.)

M. MARTIN SOLON, author of one of these volumes, is physician to the Hospital Beaujon, at Paris, an institution, comparatively speaking, very rarely visited by Englishmen, on account of its remoteness from the School of Medicine, and other more important scenes of medical action. Hence it happens that its physician is much less known on this side the channel than he deserves to be. Some experience in these matters enables us to affirm that there are no wards in the French hospitals where the student will enjoy a better opportunity of acquiring a sound acquaintance at once with diagnosis and the principles of rational treatment than in those of M. Solon. We do not mean to assert that there are not more profound masters of diagnosis in Paris, but we do maintain that in the union of the two arts—in the power of detecting and of combating disease—he is, generally speaking, superior to most of his countrymen, and entitled to a very high rank among contemporary practitioners. M. Solon's mind may be characterized as especially of a practical kind. The remaining writers, whose names are enumerated in our list, are sufficiently well known to fame to render the ceremony of introduction superfluous.

Among the works of which the titles appear at the head of this article, M. Rayer's is the only one purporting to embrace a complete view of the pathology of the kidney; we shall, therefore, take it as our groundwork in the present review, and notice, in due season, with the full detail their importance merits, the monographs devoted specially to the illustration of Bright's disease.

M. Rayer's volume opens with a very extensive section, entitled *prolegomena,* wherein, in addition to a general view of the diseases of those organs, we find the normal anatomy of the kidneys, the alterations they undergo from the progress of putrefaction, and the various changes of constitution to which the urine is subject, treated of with very considerable care. Through this part of the work we cannot, for very obvious reasons, follow our author closely, but we shall advert to such of its contents as appear likely to interest the English reader, from their novelty or practical bearing.

In imitation of his countrymen, who have made other viscera the subject of particular study, our author appears to have examined with much perseverance (and we by no means think such researches valueless, though it is certain they have not in other instances led to any useful conclusions,) the weight of the healthy kidney at different ages. The results of his enquiries are laid down in eight tables; and, though implicit confidence in their correctness may be reasonably refused, on the ground of the limited number of individual cases furnishing them, yet the following seem worthy of attention. We learn, for example, that the weight of the kidneys is never accurately the same in different subjects of the same age; that the development of those organs, though it is progressive in new-born infants, is in them subject to very remarkable individual varieties; that as a result of the foregoing propositions it is hardly possible to feel certain of the existence of renal atrophy or hypertrophy, unless in cases of very notable deviation from the normal standard; that the kidneys are lighter in females than in males; that the left is generally heavier than the right; and that in old age both organs ordinarily weigh as much as in adult age. The mean weight in thirty males, from æt. 16 to 76, was, for the right organ, ℥iv. ʒiij.; for the left, ℥ivss.; this weight is not always proportional to the size of the organs; in other words, the density of their substance is variable.

In the minute description of the anatomy of the kidneys, we observe some statements valuable from the light they throw on the pathology of certain renal affections, concerning which our knowledge has hitherto been exceedingly obscure. These anatomical details will be most usefully referred to in our account of the diseases in which the tissues described are concerned; but we may meanwhile instance the discovery of a distinct cellular membrane, between the fibrous capsule and substance of the kidney. The existence of this membrane explains the tendency to suppuration now and then observed in the locality it occupies, as also the pretty frequent occurrence of hemorrhage in the same situation, and the occasional investiture of the organs by a very thick and firm lamella—the membrane in question in a state of hypertrophy—independently of the true capsule.

The very moderate degree of sensibility possessed by the kidneys in health is familiarly known, and easily ascertainable by pressure, percussion, and other similar means. But the point has been further demonstrated in the lower animals by a number of experiments originally executed by M. Comhaire, in 1803, and lately repeated, somewhat needlessly, as it appears to us, by M. Rayer and his pupils. The kidneys were drawn out from the abdomen by these experimenters, manipulated, cut, torn, and burned with a variety of caustics, without the subjects operated on evincing signs of suffering. The practical inference from these facts is that very trifling pain or tenderness on pressure in the region of the kidney is a point of considerable consequence, and one which the medical observer should not rest satisfied without tracing to its cause; they also explain how it is that nephritis is frequently a latent disease, and in part account for the prevalent belief as to the rarity of renal inflammations.

The researches of Müller, of Straus Durckheim, and of others, on the conformation and structure of the urinary organs of animals of simple organization appear to M. Rayer unlikely to elucidate, in any marked degree, the mode of production of urinary disorders and renal lesions in the human subject; nevertheless, he conceives that, inasmuch as they have proved the fact of the secretion of urine by organs of extremely different appearance, they render intelligible the persistence of their function in kidneys that have undergone material and extensive disorganization. Now we consider it worth our while, as this view is founded on analogical reasoning, the most delusive as well as the most attractive from its simplicity of the processes whereby conclusions are arrived at, to show that it is fallacious. In truth, if such be the explanation of the fact adverted to, the inference is just, that extensive lesion of organs, possessing a closely similar structure throughout the animal series, ought not to be found to coexist in the human being with continued manifestation of activity on their parts. Now is the fact so? Clearly it is not. Do we not find that the liver continues to secrete bile when a very minute portion of it remains in a healthy condition; that the lungs exercise their wonted influence on the blood traversing them (in an imperfect manner, it is true,) when they are almost totally disorganized; that the power of thought and of reason is occasionally manifested by brains of which a large proportion is unsound? Nay, more; is it not an incontestable fact that individuals may live—and live with a certain amount of enjoyment after every important organ in their frame has become profoundly disorganized? M. Rayer's erroneous notion is an effect of the doctrine so generally held by French pathologists, that functional derangement is uniformly proportional to the organic lesions cognizable by our senses,—a doctrine which has exercised and still continues to exercise a most baneful influence on their bed-side practice.

The importance of a thorough acquaintance with the changes induced in our tissues by the progress of putrefaction is now fully acknowledged by morbid anatomists; nevertheless, only a one-sided view of the subject has as yet been taken. Enquiry has been limited to pseudo-morbid appearances, and to the signs distinguishing these from conditions originating in disease; nowhere do we find a serious and methodized attempt made (incidental allusions to them are occasionally met with) to appreciate the *pseudo-healthy* appearances, if we may be allowed the expression, which the cessation of vital action sometimes induces in really diseased parts. Yet this is a field which may be most profitably cultivated by those desirous of giving the study of morbid anatomy all the precision of which it is capable. Our author does not swerve from the ordinary plan in his general view of putrefactive changes in the kidney, and enumerates alteration of colour, separation of the fibrous capsule, emphysema, and the effects of desiccation and of maceration as the chief points for consideration.

The phenomenon of hypostatic engorgement of the cadaveric species is of such regular occurrence in the kidneys, that, according to M. Rayer, whenever their anterior is more injected than their posterior portion, in subjects who have been laid, as is usual, on the back, this condition is to be ascribed to morbid action. One of the most remarkable effects of imbibition of blood, when considerable and affecting *both* substances, is, we are told, the assimilation of their respective colours, a condition which has been very frequently mistaken for the effect of disease. The separation of the fibrous capsule is, in cases where abdominal putrefaction advances with rapidity, a very usual phenomenon, and is directly produced by the softening of the cellular membrane already alluded to. This separation of the capsule does not occur spontaneously in kidneys affected with chronic inflammation, in consequence of the thickening and abnormal adhesion of the proper cellular membrane to its internal surface.

There are some peculiarities in the cadaveric emphysema of the kidney. That it should rarely exist if there be little blood in the vessels, and not be developed at all when they are almost free from that fluid, is sufficiently intelligible; but why it should be absent, as M. Rayer avers is the fact in some cases, when the organs are perfectly rotten, disengaging a strong odour, of greenish hue externally, while both substances are saturated with blood, is not easily explicable.

We are given no original instruction on the method of distinguishing putrefactive from morbid softening; and the description of the changes to which the *colour* of the organs is liable increases the perplexity one is likely to feel pretty often in deciding on its causation. So rapid are the alterations of tint, that M. Rayer has seen an artist sadly puzzled to catch the original colour. The only way of avoiding effectually this source of error consists in examining and noting down the colour of the surface the moment the fibrous capsule is removed.

Maceration has, as might be foreseen, the effect of rendering the peculiarities of some morbid conditions unusually distinct, those of others more obscure, and of depriving a third set of all their characteristic qualities. Among those rendered more apt for examination are the granulations in the affection described by Dr. Bright, as that observer had himself stated. The impregnation of the surrounding cortical substance with water renders it semi-transparent, and so throws out the dull, milky hue of the granulations. According to M. Rayer, those bodies are usually more distinctly visible at the inferior part of the right kidney than elsewhere; this he attributes to putrefaction commencing earliest in this portion of the organs.

M. Rayer's classification of renal diseases is comprehensive, and gives fair evidence of his possessing a spirit of order and method. There is no possible morbid condition to which the kidneys are liable that does not here find its particular place; but at the same time we must remark that it can hardly be considered correct to class congenital anomalies and mechanical injuries as diseases; nor is it easy to understand why certain abnormal states should occupy the ground they do. Why, for example, hyperæmia and anæmia should follow not precede inflammations, or why gangrene should be separated widely by totally dissimilar conditions from those same inflammations. Possibly the concluding volume may explain, though it will be difficult for it to justify this departure from natural arrangement. We subjoin a table of the principal heads, omitting all their numerous subdivisions.

**A. Diseases of the Kidneys.**

1. Wounds, Contusions, Lacerations.

2. Inflammations — Nephritis, Pyelitis, Pyelonephritis, Perinephritis, Renal Fistulæ.

3. Hemorrhages.—Idiopathic, Symptomatic, Endemic.

4. Hyperæmia. Anæmia.

5. Hypertrophy. Atrophy.

6. Tumours formed by retention of urine — Hydronephrosis, Kysts.

7. Diseases of elementary tissues —

   - Veins,

   - Arteries,

   - Lymphatic glands and vessels,

   - Nerves.

8. Morbid tissues and products — Homologous, Heterologous.

9. Foreign bodies — Animate, Inanimate.

10. Gangrene.

11. Anomalies in —

    - Number,

    - Situation,

    - Configuration.

**B. Special alterations of the urinary secretion.**

1. Quantity —

   - Suspension, Diminution, Augmentation,

   - Diminution of one or several natural principles,

   - Increase of some natural principles.

2. Composition — Presence of adventitious principles, whether of morbid formation or introduced from without.

3. Mode of excretion. Uroplania.

The same arrangement is followed for the morbid states of the ureters and supra-renal capsules as for the kidneys themselves.

A glance at this table will suffice to show the practical sagacity evinced by M. Rayer in abstaining from a regular system of generalizations on a set of diseases so utterly dissimilar in cause, nature, course, and termination. The few he indulges in, especially those regarding the relations of renal affections to those of other organs, are interesting and apparently well founded ; but more important matter for notice is elsewhere furnished in abundance.

In proceeding to examine a patient whose urinary organs are suspected to be the seat of disease, inspection, manual examination, and percussion of the renal regions should first be had recourse to ; of this all will admit the truth ; but so important is it for practitioners to act on the admission, that we may be permitted to state in a few words some of the diagnostic information derivable from those methods of exploration. Thus, for example, in certain cases of cancerous diseases of the kidney, of pyelitis (inflammation of the pelvis and calices), of hydronephrosis (tumour formed by retention of urine), &c., those regions become manifestly prominent or otherwise deformed ; whether the altered shape really depend on a renal affection, or, as may with almost equal *primâ facie* probability be the case, on an intestinal, hepatic, splenic, or ovarian tumour, or subperitoneal abscess, &c., must be determined by other means, among which manual examination first presents itself. By this the degree of sensibility of the kidneys, and the extent, form, direction, mobility, and resistance of visible intumescences may be ascertained. The importance of any notable tenderness has already been adverted to; we may be guided in regarding such tenderness as dependent on the kidney in contradistinction to the surrounding organs, if it be most distinctly marked posteriorly, "as pain in the corresponding part of the peritoneum, large intestine, liver, or spleen is in general most felt by the patient when pressed or percussed on the antero-lateral part of the flanks." Percussion will further aid in making out the origin of tumours in the lumbar regions; but the dull sound elicited by that process may, it must not be forgotten, be due to enlargement of other solid organs; the commemorative symptoms, as well as those present at the time of examination, must therefore be diligently scrutinized. It is needless to dwell on the importance of a careful and thorough enquiry into the state of the urine; the normal or abnormal characters of that excretion may in certain cases at once settle the question of the absence or presence of serious disease in the kidney, a fact with which the labours of Prout and Bostock have long familiarized English practitioners. But, as our author very justly remarks, the whole process of examination we have thus briefly noticed will not, even if extended to the ureters and bladder, prostate and urethra, and followed up by an enquiry into the causes of the affection, be sufficient, in all cases, for our purposes of diagnosis; the state of the constitution must be well ascertained before we can safely determine that point, or the mode of treatment advisable. Two instances will be enough to show the practical truth of this statement: in many cases of nephritic colic, and lithic acid deposition, it is the hereditary or acquired arthritic diathesis that the physician must labour to remove; the mere exhibition of alkalies would, in such cases, relieve a symptom, but nothing more; again, in cancerous or tuberculous diseases of the kidney, it is manifest that our therapeutic measures must be directed against the vitiated state of constitution giving rise to those disorders.

The next 200 pages of M. Rayer's volume are devoted to general considerations on the urine in the state of health; on the different characters it assumes from differences in respect of diet, age, &c.; on the modes of examining it, the modifications of its natural constituents, as well as the accidental introduction of foreign principles; on the clouds, sediments, and cremors occurring in it; on the phenomena of its putrefaction, and the relations subsisting between its morbid states and those of the blood and other fluids. We are bound to express our most unqualified approbation of this part of the work; it gives evidence of the soundest and most extensive erudition, as well as of a degree of practical familiarity with the subject treated, not easily to be surpassed. It is pleasing to observe, from its contents, that the labours of our own countrymen have very clearly had the most important share in giving its existing perfection to urinary pathology; while it is not a little creditable to M. Rayer, living as he does in a country where the Practical stands extremely low in general estimation, to have comprehended the sterling excellence of our contributions to useful medicine.

M. Rayer shows that, historically considered, the progress of knowledge respecting the urine may be divided into three periods: the medical, the chemical, and the medico-chemical. The first commencing with

Hippocrates, extended to the time of Van Helmont, and was marked by the exclusive attention given to the outward appearances of the urine. The second, of which M. Rayer is probably correct in fixing the origin with Van Helmont, has been chiefly illustrated by the analytic labours of Bellini, Willis, Boerhaave, Rouelle, Scheele, Wollaston, Berzelius, Marcet, &c., and cannot be said to have yet terminated. The medico-chemists made their first appearance in the person of Cruickshank, who set the example of studying the urine especially in its relations to morbid states of the system, basing his researches on accurate chemical analysis; he has been worthily followed by Nysten, Prout, Brande, Henry, Bostock, Christison, Rees, and many others. Further, a new or rather a revived method, for it appears to have originated with Leeuwenhoeck, of studying urinary deposits, namely, with the microscope, has been of late pressed into the service by M. Donné; and, judging from the valuable results already obtained by that gentleman, and MM. Vigla and Quevenne, the microscopical is likely to exercise quite as important an influence on urinary pathology as any other mode of investigation.

The results of M. Rayer's experiments on the urine of infants, performed in conjunction with the distinguished chemist Guibourt, do not accord with the opinions expressed on the subject by a variety of writers. Thus, instead of finding the urine of infants muddy at the moment of emission, they distinctly ascertained, as they state, that it is then perfectly transparent, and further, that it does not become turbid more rapidly than that of adults. The urine of infants at the breast they found to be neutral, and without urinous smell when voided, though it evolves that peculiar odour during the process of evaporation; no appreciable formation of nitrate of urea takes place when its extract is treated with nitric acid. The urine of a healthy infant, three years old, furnished, on the contrary, a full proportion of urea, but none of the benzoic acid, which almost all chemical writers, on the authority of Fourcroy, enumerate among the constituents of the renal secretion in children. We think it right to produce this statement of our author, especially as it is confirmative of Dr. Prout's opinion on the point. M. Rayer also declares his disbelief of the current notion that an excess of phosphate of lime is characteristic of the urine of persons advanced in years; his incredulity seems to rest on a very limited number of experiments.

The subject of specific gravity is examined at some length by our author;—without containing anything positively new, his summary embraces all the facts regarding it, which are firmly enough established to be relied on in practice. The different degrees of confidence with which this character of the urine is regarded by different observers as a diagnostic sign is extremely remarkable; while some reject it altogether as the most deceptive of guides, others look upon it as furnishing most valuable indications. Those who adopt the former mode of thinking (instance M. F. d'Arcet who has made a series of experiments on the subject, whence it would result that the specific gravity of normal urine varies from 1,001 to 1,060)* have evidently done so out of mere disgust at the difficulties attending the proper appreciation of the various circumstances.

* L'Expérience, No. 55, Aug. 1838. This paper deserves to be read.

capable of influencing the character in question; the holders of the latter have unwisely generalized the results of a few favorable cases. The truth is, that diseased conditions do exist, but they are limited in number, wherein it is possible to obtain useful information from the variations in density of the urine. The first point to be ascertained is the average healthy specific gravity: now the multitudes of circumstances influencing this, such as temperature, the quantity and quality of food and drink, the state of the bowels and of the cutaneous and pulmonary exhalations, the period after exercise and meals at which the urine is voided, the length of time it has remained in the bladder, as well as that which has elapsed between the emission and examination, the hydrometer employed, &c. render it doubtful, whether any one will be found willing to undergo the vast labour such an investigation would require. Meanwhile, whose approximate estimate is to be regarded as the most accurate? Are we to accept as correct the calculation of Dr. Prout, which fixes the mean density at from 1,010 to 1,015; or trust to that of M. Rayer, who gives us the number 1,018, or to Drs. Bostock and Martin Solon, who consider 1,020 nearer the real mean; or to adopt the opinion of Drs. Gregory and Christison, who assert that it is so high as 1,024—1,025? Trifling as these differences may appear, they are capable of causing real discrepancy of opinion as to the morbid or normal condition in this respect of certain specimens of urine: a density of 1,020 will show that fluid to be normal in the patients of Dr. Bostock; from five to ten degrees above the healthy standard in those who consult Dr. Prout, and five or six below it in individuals who chance to be under the care of Dr. Christison. Now, in illustration of this, we may state that the last-named observer and M. Rayer do not accurately agree respecting the condition of the density of the urine in the early stage of Bright's disease, Dr. Christison declaring that it is moderately lower, the French writer that it is occasionally superior—as is the case in the only examples given in his accompanying table of densities—to the healthy mean. This want of accordance arises in some degree at least from their different notions respecting that mean.

The sources of inaccuracy increase materially in various conditions of disease; the density of the fluid undergoes marked changes, which we know not how to explain in such cases. An interesting example of this is given by M. Rayer, in the case of an individual affected with chronic pyelitis; the urine of this man marked 1,008·4 and 1,016·4 at eight o'clock in the morning on two consecutive days, though no change had taken place in the conditions known to affect the specific gravity of the fluid. Again it varied from 1,007 to 1,018 from one day to another in a patient of M. Solon affected with Bright's disease; no cause for this peculiarity was discovered.

From these considerations it results that, in order to possess diagnostic value, the variations in specific gravity must be at once well marked and persistent; in diabetes and in the advanced stages of Bright's disease, where all are agreed as to the real utility of this sign, it is thus characterized. M. Rayer gives a table of the densities observed in a variety of local and general, trifling and serious disorders; we confess we do not see its value, for it expresses the mean results of *five* examinations only in each disease.

We shall not pursue this subject further for the present than to state that the specific gravity of the urine may be lower than that of the liquids used as drink. Thus M. Rayer informs us, that one of his patients, who drank a bottle of artificial Vichy waters every morning, frequently voided transparent urine in his presence, of lower density, even when cool, than that mineral solution.

Some points in M. Rayer's section devoted to the history of that very important principle, urea, deserve notice. Amongst these is the method—valuable from the facility of its employment—of estimating roughly the proportion in which it is present in any given urine: a drop of the fluid is poured on a plate of glass; in warm weather this small quantity is in a few minutes sufficiently condensed by spontaneous evaporation (in the winter the aid of gentle heat is required), to allow of the formation of a magma or crystalline mass on the addition of a similar quantity of nitric acid. Examined under the miscroscope this is found to be composed of brilliant acicular crystals of nitrate of urea.

Respecting the variations to which the proportion of urea is liable in disease, our author's experience has convinced him of the rarity of cases attended with an excessive formation of that principle, and, on the contrary, satisfactorily proved the frequency of morbid states in which its proportion falls below the healthy standard; the common occurrence of the latter he is probably right in ascribing as much to the abstinence and lowering regimen enforced for their cure, as to the direct influence of the diseases themselves. But there is another cause of diminution in the proportion of urea discoverable by analysis, which has only been of late years suspected to exist; we allude to the decomposition presumed to be undergone by the principle in question under certain conditions. The notion of its possibility seems to have originated with Wöhler, who remarks that "the transformation of urea into cyanic acid and carbonate of ammonia, and that of uric acid into urea and cyanic acid, may at a future period become important in a physiological point of view, and throw light on the origin of certain diseases and abnormal sediments of the urine."* The plausibility of this view is recognized by M. Rayer, for he inclines to attribute the deficiency of urea and lactic acid, (not uric, as erroneously printed in his book,) detected by MM. Cap and Henry† in a specimen of viscous urine, to the occurrence of such decomposition, on the ground that viscidity is often the result of the action of ammonia on mucus and pus, and may have been so induced in the case adverted to. Further on, he speaks more unreservedly; and affirms that in the majority of cases of inflammation of the mucous membrane of the pelvis and

* Journal de Pharmacie, vol. xvi. p. 300.

† On detecting the deficiency of these principles, it occurred to the above-named experimenters, that an artificial combination of them might be administered advantageously in certain conditions of the urinary passages. Accordingly, they set about the manufacture of the salt, and succeeded in procuring it by decomposing oxalate of urea with lactate of lime: they promise to make public the result of its exhibition. It is right to mention that these chemists suppose urea to exist in the urine as a lactate, and that to its state of combination is due the difficulty of separating it from the fluid without the aid of nitric acid. They represent that when they had removed the free lactic acid from the urine with an excess of hydrate of zinc, they were enabled easily to obtain natural lactate of urea, crystallized, and perfectly identical with specimens of the salt directly prepared.—For further details we must refer to *L'Expérience*, No. 39, vol. i. p. 621.

bladder, consequent on retention of urine in those cavities, the urea undergoes decomposition, and that the carbonate of ammonia resulting therefrom must be taken into account in estimating its primitive proportion. He even considers it probable that, in a certain share of the cases in which the urine possesses an alkaline reaction, independently of the action of medicines, immediately on being voided, the quantity of urea obtained by analysis will be less than that primitively secreted. Nevertheless, he hardly appears firmly persuaded that alkalescence is in such cases induced subsequently to the act of secretion. He elsewhere, indeed, (in alluding to the fact ascertained by M. Guibourt, that urine which has remained in the bladder for a certain period, and proves alkalescent at the moment of emission, contains proportionally less urea than the healthy fluid,) remarks that, "as urine which is not exposed to the air, does not putrefy even at a temperature of 32° R., it is probable that that fluid, when long retained and containing carbonate of ammonia, owes this peculiarity to an act of secretion rather than to putrefaction." But the experiments on which this statement is founded, were, it must be remembered, performed on healthy urine; whereas, that spoken of by M. Guibourt had been retained in the bladder with blood, mucus, pus, or other principles acting as alkalies, and capable of promoting the decomposition of urea: the two kinds may be demonstrated to comport themselves differently, when placed in hermetically sealed vessels. On the other hand, a certain number of facts have been observed, tending to show that urea may be converted into carbonate of ammonia during the act of secretion; or, if this mode of expression be objectionable, that the latter may be elaborated by the kidney instead of the azotised principle. The strongest fact of this kind—its conclusiveness, as admitted by Drs. Graves and Willis, may be doubted—is one published some years past by the former of these physicians. The patient in this case was made to empty his bladder thoroughly, and half an hour after to discharge the urine that had accumulated in the interim; this last was instantly analysed and found to contain an abundance of carbonate of ammonia, but not a trace of urea.

While on this subject, we may state that M. Rayer completely denies the correctness of the common notion as to the alkalescence of the urine in typhoid fevers. In *fifty* cases examined at the most marked periods of the disease, he only found it alkaline *twice;* even when retention of the contents of the bladder occurs in this affection, he affirms they are almost invariably acid. How are we to reconcile these very confident declarations with general belief,—how especially with Dr. Willis's experience, which authorizes him in sometimes basing a flattering prognosis on returning acidity, even in cases where nothing else betokens improvement?*

We shall scarcely be accused of travelling out of the region of purely practical disquisition, if, with M. Rayer, we enter slightly into the question of the cause of the state of solution in which lithic acid exists in healthy urine. Various chemists have each offered a different explanation of the well-known difficulty attending its dissolved condition therein: Dr. Prout's theory is admitted to be the most plausible and ingenious yet advanced.

* On Urinary Diseases, p. 128.

This gentleman, as is well known, conceives that the acid in question is not present in a free state, but in combination with ammonia in such proportion as to form a superlithate of that base. Now, as that salt possesses a comparatively high share of solubility, if the notion of its existence be correct, the solution of the lithic acid ceases to be a mystery. It is needless to recapitulate Dr. Prout's well-known arguments, but we shall lay before our readers such evidence against and in favour of the combined condition of the acid, as more recent enquirers have supplied.

M. Donné, whose interesting contributions to microscopical studies have more than once been noticed in this Journal, espouses the opinion of Dr. Prout. He maintains that lithic acid is always found in the urine crystallized, and not in an amorphous powder. On the other hand, M. Quevenne, while he admits the possibility of their occasionally containing a certain share of urate of ammonia, affirms that all the amorphous sediments of acid urine observed by him, whether they were gray, pink, or laterititious, were essentially composed of lithic acid in union with animal matter; and that, when recent, they gave but very slight indications of the presence of ammonia. "No doubt," he adds, "if they are examined some time subsequent to emission, the alkali may be very distinctly perceptible, and may even be found to have given rise to the formation of blackish globules consisting of lithate of ammonia." These globules occasionally appear a few hours after the urine has been discharged, a circumstance which, as M. Quevenne admits, justifies a suspicion of a considerable quantity of lithic acid having been in combination with bases at the moment of emission.

M. Donné adduces the two following strong facts in favour of Dr. Prout's theory: first, the sediment of acid urine redissolves, exactly as lithate of ammonia is known to do, if the fluid be boiled, whereas lithic acid once crystallized cannot be so dissolved; secondly, the action of a weak acid, such as the nitric diluted with from eight to ten times its weight of water, has no effect on lithic acid, but decomposes the amorphous sediments and transforms them into perfect crystals of that acid. M. Quevenne, however, is not without his answer to these allegations; both facts, according to him, admit of a different explanation from that proposed by his opponent. Suppose, he argues, the amorphous powder to be composed of lithic acid in the condition of a hydrate, and there will be no greater difficulty in accounting for its solution by ebullition than for that of a lithate; and again, whether the acid be combined with ammonia, animal matter, or water, the production of crystals of lithic acid, by the action of a weak agent of the same class, is equally intelligible. M. Rayer professes himself, as we think, on very just grounds, best satisfied with the reasoning of M. Donné.

Passing over a variety of useful matter, as our limited space obliges us to do, we arrive at the section treating of the organic products accidently introduced into the urine. It results from recent microscopical investigations—the discovery originated, we believe, with M. Raspail—that the genito-urinary epithelium, as well as that of other mucous membranes undergoes continual desquamation in the natural state, and that in certain diseased conditions, this physiological process acquires morbid activity. Examined under the microscope, the resulting scales are found to be of the extremest delicacy, and transparent, except where marked by fine lines; some points appear to correspond to minute openings; their form is irregular, their border ragged. In the female, the use of the catheter will decide whether they come from the pudendum or the proper urinary passages: that their separation does really take place in the most remote parts of those passages is proved by their being discoverable in the urine found in the calices and pelvis after death.

Among the adventitious matters appearing in the urine, the blood in substance, or its constituent principles individually, hold the first rank in point of importance. The researches which have of late years been made in this country, and followed up with vigour in France, further show that not one of these claims so large a share of the practitioner's attention as *albumen.* Our readers will therefore not be surprised, especially when they consider its comparative novelty, if we enter at some length into the subject of albuminous impregnation of the urine. Our remarks shall for the present be strictly of a general kind.

The presence of albumen in the renal excretion may be established by the majority of reagents which serve for its detection in pure water; but the evidence of many of these cannot in the case of the urine always be trusted to. The combination of three characters, coagulability by heat and by nitric acid, and non-precipitation by acetic acid, affords and alone affords incontestable evidence of the presence of albumen. The necessity of the coexistence of these characters is frequently overlooked, and deserves illustration at our hands. Urine, containing milk or casein, whether naturally or by artificial introduction, (a trick which, in these days of extraordinary attention to urinary pathology, it is reasonable to expect may soon become a favorite resort of *malingerers**) will coagulate by heat, and precipitate with nitric acid; but, unlike albuminous urine, will also coagulate on the addition of acetic acid. Again, when albuminous urine is alkaline, no matter how it has become so, whether by long standing or otherwise (hence the importance of making our analysis as soon as possible after emission), it does not ordinarily lose its transparence under the action of heat, unless the quantity of the foreign principle present be very considerable, and even in this case simply assumes a milky tint. The addition of a certain quantity of nitric acid will cause instantaneous coagulation; but, according to M. Rayer, it is a mistake to suppose that the presence of alkalies will always cease to influence the coagulation of the albumen by heat the moment the fluid containing them is *neutralized* with an acid. He declares that he has ascertained that nitric acid, in the proportion of a drop to a drachm, will not ensure turbidity under ebullition; but that, on the other hand, precipitation takes place in alkalescent albuminous urine with or without the aid of heat, if the quantity of acid be increased to "several drops." Curious to discover the cause of the non-coagulation under these circumstances, MM. Rayer and Guibourt made some experiments on white of egg dissolved in water. Their researches have not been successful in this point of view, but have so far established the conditions under which it occurs, as to show that nitric acid in small quantities, (i.e. from $\frac{1}{72}$ to $\frac{1}{100}$ of the tested fluid) acetic in large, and phosphoric acid in small or large proportion, deprive albumen of the property of being coagulable by caloric, but not of being precipi-

* It has already been had recourse to by at least one of M. Rayer's patients.

table by nitric acid. It follows, according to M. Rayer, as a direct consequence of these experiments, that, as specimens of urine containing at once albumen and a little phosphoric acid may be met with, nitric acid is a more certain and generally applicable test for that principle than heat. Dr. Christison's experience does not quite accord with that of M. Rayer on this point, for he has "found that sometimes even nitric acid added in excess did not separate albumen which had been present in large quantity." The experiments of the French observers were not conducted on perfectly correct principles; Dr. Wells long since showed that the effects of reagents are not the same on albumen contained in water and in urine.

It must also be remembered that, if alkaline urine be rendered turbid by heat, the loss of transparence is usually due to the precipitation of phosphates, as is proved by its complete restoration on the addition of nitric acid. For this fact we are, we believe, indebted to Mr. Rees.

If the urine be muddy, from the deposition of lithic acid and the lithate of ammonia, heat will, according to Dr. Christison, remove the turbidity by dissolving those compounds: we doubt this as respects the first of them. Mucus, if present, will not disappear under the action of heat, and, as it causes muddiness, should be removed by filtration before the test is employed.

On the other hand, precipitation by nitric acid alone will not prove the matter thrown down to consist of albumen; such precipitate may be composed of lithic acid or lithate of ammonia. Here has, doubtless, been a source of very frequent error, and, according to M. Rayer, the additional one has more than once been committed, in consequence of the precipitate of lithate of ammonia being soluble in an excess of acid, of attributing that character to albuminous coagula. All this we are somewhat at a loss to reconcile with the declared superiority of nitric acid as a test; but, however this may be, M. Solon has made some experiments which show that M. Rayer's denial of the possible redissolution of albumen in an excess of precipitant requires qualification. He found that the coagulum, obtained from an artificial mixture of two drops of serum and one ounce of normal urine, increased in quantity, until fifteen or twenty drops of nitric acid had been added to it, but disappeared completely when the dose of reagent had amounted to thirty drops. But this redissolution, even where the proportion of albumen is very small, requires, at least, twenty-four hours for its accomplishment; hence, in all probability, the difference of opinion of the two observers. We have repeated M. Solon's experiment, and vouch for the correctness of the result announced: the same effect takes place in the coagulable urine of Bright's disease.*

* We shall employ this phrase throughout the present article to designate the morbid state of the kidneys, made known to the profession by Dr. Bright, because it involves no erroneous or disputable position, and because we do not share Dr. Christison's polite scruples (Preface, p. xiii.) as to the propriety of its adoption. *Granular degeneration of the kidneys* is so utterly incorrect a term, that we cannot understand the latter gentleman's employment of it, more especially, as his doing so tends to keep alive the error into which he presumes, with what justness we shall presently see, Professor Forget had fallen, that of supposing granular deposition an essential part of the renal lesion. M. Solon's neologism, *albuminuria,* is inadmissible as a title for the disease, because, literally interpreted, it is applicable to a symptom only, and this one which occurs in various conditions; but the laxity of that gentleman's use of the word is really sur-

The matter thrown down by nitric acid may at once consist of the proximate principle in question, uric acid and urate of ammonia: microscopic inspection will, in such cases, prevent error, by disclosing the lamellar, corrugated, and peculiar appearance of albumen, crystals of lithic acid, and an amorphous powder, convertible into similar crystals by nitric acid (lithate of ammonia). The proportions of the three ingredients may be ascertained by acetic acid and ebullition. Again, if albuminous urine be red-coloured, from the presence of hæmatosine and globules of the blood, nitric acid, in a great measure, discolours it by precipitating all the foreign principles together; the microscope will detect the globules, either in the urine or imprisoned in the flakes of albumen.

The different aspects of the coagulum depend, in a great measure, on the quantity of albumen present, and this is exceedingly variable. Barely discernible by the usual tests in some cases, in others it has been known to constitute $\frac{27}{1000}$ by weight of the mass of urine; where the proportion is so low as one part in a thousand, ebullition and evaporation should be prolonged for a considerable time, a precaution that has, we fear, frequently been neglected. If it be particularly desirable to learn the precise quantity of albumen present, this may be effectually done by taking the coagulum, obtained by heat, washing it in alcohol, drying and weighing it, and then subtracting the amount from the total weight of the urine employed. Such an accurate estimate as this, however, is not absolutely called for in practice; a simple statement of the quantity of coagulum formed, may be accepted as a satisfactory substitute, and we so perfectly agree with Dr. Christison, as to the scientific and practical benefits which must follow, were practitioners "to employ some common nomenclature for the different degrees of coagulability," that we reproduce the scale he has proposed, in the hope of its being generally adopted, until a better be brought forward.

"1. *Gelatinous by heat.* 2. *Very strongly coagulable,* where a precipitate distinctly separates by heat, and yet occupies, in twenty-four hours, the whole, or nearly the whole, fluid. 3. *Strongly coagulable,* where the precipitate, in twenty-four hours, occupies half the volume of the fluid. 4. *Moderately coagulable,* where it occupies a fourth of the fluid. 5. *Slightly coagulable,* where it occupies an eighth of the fluid. 6. *Feebly coagulable,* where it occupies less than an eighth of the fluid. 7. *Hazy by heat,* where the urine becomes cloudy, but does not form visible flakes a few seconds after being boiled." (p. 44.)

It seems unnecessary to dwell at any length upon the numerous other reagents proposed for the detection of this principle; as they are all confessedly inferior in accuracy, and not superior in facility of employment to those described, it will be enough to enumerate them, and refer the curious to the original volumes for further detail. They are tannin, creasote, alcohol, ferrocyanate of potass and acetic acid, bichloride of prising; it is successively made to mean *dropsy caused by disease of the kidneys* (Title-page, &c.); *dropsy with albuminous urine, caused by disease of the kidneys* (passim); *dropsy with albuminous urine, without renal lesion* (p. 109, &c.); and simply *albuminous urine* (passim). In the latter, its true acceptation, we shall ourselves employ it. *Albuminous nephritis,* the name proposed by M. Rayer, may be founded on correct views of the nature of the disease, but we are unwilling to avail ourselves of it, because we are not, at present, entitled to assume, as certain, that an inflammatory condition is invariably the starting point of the disease, though we admit the extreme probability of the fact; besides albuminous urine occurs in different varieties of renal inflammation.

mercury, and alum. A character of albuminous urine, which may sometimes be usefully consulted, is its frothing considerably more than the healthy fluid, when blown into or even shaken; the bubbles produced remain unusually long without bursting.

Albuminous urines differ from each other in a variety of respects. If acid, when voided, and containing but little fat and mucus, they may be perfectly transparent: the fluid may, in other instances, (as is frequently the case, for example, in the early stage of Bright's disease,) be of a deep red tint, from the presence of the colouring matter of the blood, or assume a straw-coloured or pale greenish-yellow tinge, like non-clarified whey, as in the more advanced periods of the same affection. Again, the quantity of fluid discharged from the bladder is very variable: in Bright's disease alone it ranges between a quantity superior to the normal standard of excretion and two or three ounces. Dr. Wells states, but (as he adds, with most commendable respect for accuracy,) "on the authority of patients and nurses," that dropsical patients sometimes void six, eight, or ten pints of serous urine daily. But the most important point of difference in these abnormal urines respects their remaining normal or adventitious constituents. The proportion of urea and salts may be increased, natural or diminished. M. Rayer instances purpura as an affection in which the first condition is observed, and the advanced stage of Bright's disease as furnishing an illustration of the last. With respect to the detection of urea in albuminous urine, the process of Mr. M'Gregor,* for the discovery of such portion of it as may be precipitated in the substance of the coagulum, is sufficiently accurate for all practical purposes; but the residual urine must also be carefully analyzed, for, as MM. Rayer and Guibourt have found, the quantity of urea therein contained may be "more than one hundred times greater than that obtained by the process in question from the coagulum of the same specimen of urine."

In the albuminous urine of acute affections there is an abundant precipitation of lithate of ammonia: in chylous urine the albumen is associated with fatty matter: in others, mixed with pus, the colouring materials of the blood, &c. Hence arise numerous varieties in point of specific gravity; M. Rayer has found it so high as 1,032, in the commencing stage of Bright's disease; Professor Christison so low as 1,004, or probably even 1,001·5, in the advanced period of the same affection.

Dr. Wells, the only person apparently who has examined this point, generally found albuminous urine to possess the saline and bitter taste of that which is healthy, "though sometimes in a less degree, than even its copiousness would account for."

In considering the abnormal character of albuminous impregnation, the question very naturally arises, whether the adventitious principle be vicarious of any of the healthy constituents of the renal secretion. This question has not yet, however, been examined in a general way, with reference to all the various cases in which such impregnation may occur, and seems to have met with attention in regard to Bright's disease alone. The albumen discharged in that disease has been by some regarded as succedaneous of the urea, which is presumed to be proportionally deficient. A careful revision of the few cases yet published, with the

* Medical Gazette, May, 1837.

necessary details on this point, teaches us to doubt the correctness of this notion. It is no doubt true that urea is deficient in pale-coloured albuminous urine of low specific gravity, and that in the deep-coloured variety of high density it is often present to a considerable amount; but it does not thence follow that it exists in the inverse ratio of the abnormal ingredient, and that the contrary is more likely to be the truth appears on the evidence just alluded to. It may be well to quote the statement of Dr. Christison, in reference to this topic; though published so far back as 1829 it is accurately applicable, as he informs us, to all his subsequent experience.

"In most of the cases I have seen, where the urine was very pale, of very low specific gravity, and deprived of the greater part of its urea, the quantity of albumen was small, never exceeding three parts and a half of dry albumen per thousand, while in the cases where the urea was considerable in quantity, the albumen was also considerable, being in the former so high as ten or eleven parts in the thousand. Besides, the secretion of albumen may be nearly or entirely prevented by proper treatment, without the secretion of urea being restored." (p. 52.)

M. Rayer has shown the advantages derivable from microscopic inspection of the urine under consideration; such examination is indispensably necessary for the recognition of the globules of pus, mucus, or blood, and of the particles of lithic acid and lithate of ammonia, which are frequently thrown down in union with the coagulum.

Albumen has not been admitted by chemists generally to a place among the normal constituents of the urine; and since attention has been particularly directed to this subject, no facts seem to have been elicited proving error in their views. It is certainly true that this failure in its detection may depend not on the total absence of the principle, but either on its minute proportion, or on its assumption of properties distinct from those it possesses when mixed with other fluids, in other words, on the imperfection of the existing methods of analysis: when Liebig, Dumas, and Christison admit that organic chemistry is in its infancy, it is wisdom to doubt. Cautious assertion is here the more requisite, as we find Henry, at least, enumerating this principle as one of the twenty-one constituents of healthy urine; Chevallier, too, is of opinion, as we are informed by M. Piorry,* that it invariably exists therein in very minute proportion. Dr. Osborne, besides, affirms, but this is mere affirmation, that some individuals constantly secrete urine, coagulable by long-continued ebullition, and fancies the peculiarity caused by a habit of drinking sparingly, whereby a concentrated urine is produced.†

In practice, however, we are fully warranted, on the evidence of repeated examinations, made with a view to the settlement of this point, in asserting that the liquid, normally elaborated by the kidneys, contains no albumen. And, on the other hand, it is indubitable that the fluid discharged from the bladder is found, in a considerable variety of local or general derangements of the system, to be impregnated either temporarily or more or less persistently, either slightly or abundantly, with that proximate principle. It is, perhaps, impossible to establish any classification of these derangements which shall embrace under the same head, all those resembling each other in most essential particulars, and separate such as are marked by special characters; but we venture to

* Traité de Diagnostic, &c. i. 36.

† On Dropsies, p. 8. 2d Ed.

propose the following, provisionally, as the least objectionable in the existing state of our acquaintance with the subject. A most important distinction between albuminous urines, practically considered, and one which of course exists in nature, is their being thus impregnated during the process of secretion, (which might depend either on a morbid state of the blood, or on a defective exercise of its secretory function on the part of the kidney,) or simply by subsequent admixture; but as it seems hardly possible to decide, in many cases, to which of these categories albuminous urine belongs, we have not adopted it as the basis of our classification. Our fourth class is a full one; some of our readers may probably contend that it should be still fuller.

ALBUMINURIA may be caused by

- A. *An abnormal condition of the blood, dependent on*

  - Scurvy,

  - Purpura,

  - Hemorrhagic eruptive fevers,

  - Absorption of pus?

  - ——————albuminous or dropsical effusions?

- B. *Lesions of genito-urinary apparatus.*

  - *a.* Functional.

    - Essential hæmaturia,

    - Diabetes,

    - Secretory excitement of urinary organs and passages, produced by .............................

      - Articles of food.

      - Medicinal agents.

    - Active renal hyperæmia.

  - *b.* Organic.

    - 1. Which cause the foreign admixture during the act of secretion.

      - Acute and chronic simple nephritis,

      - Pyelitis,

      - Bright's disease.

    - 2. Which cause impregnation subsequently to the act of secretion.

      - Blood, thrown out in cases of

        - Contusions, wounds,

        - Calculous pyelitis,

        - Cancer of kidney,

        - Fungous tumours,

        - Acute cystitis.

      - Tubercle,

      - Encephaloid,

      - Strumous matter,

      - Pus ... e.g. in cases of prostatic abscess,

      - Muco-pus, in catarrhal inflammation of mucous membrane of urinary passages, especially of the bladder.

- C. *Accidental admixture of healthy genito-urinary albuminous products.*

  - Semen,

  - Prostatic secretion,

  - Catamenial fluid.

- D. *Doubtful cause.*

  - Acute febrile affections,

  - Hysteria?

  - Scarlatina,

    - Primary fever,

    - Succeeding anasarca,

  - Gout?

  - Chronic diseases independent of renal lesion,

  - Chylous urine.

We proceed to make a few remarks upon the majority of these conditions, in so far as they may be productive of albuminuria. We beg to premise that we do not (except where the contrary is expressly stated) vouch for the authenticity or correct observation of the facts on which their claim to be so ranked rests ; yet it is just to add that the zealous candour with which the subject has been investigated by the majority of observers leaves but little room for well-grounded scepticism. Further, we desire it to be understood, that we designedly avoid, for the present, any discussion respecting the degree of faith to be attached to an albuminous condition of the urine as diagnostic of Bright's disease. When treating of that affection we shall carefully examine the points, wherein the albuminuria symptomatic of its existence differs, as it is maintained, from that occurring under other local or general conditions of the economy.

A. That certain morbid states of the blood might lead to albuminous impregnation of the urine seems admissible, *à priori;* and experience has established the possibility of their having such influence. M. Rayer states that he has found that the albumen and globules of the blood pass occasionally into the urine in cases of scurvy, purpura, and hemorrhagic fevers, while the fibrine diminishes in the vessels, and the fluid portion becomes infiltrated into the cellular tissue, or exhaled on the surface of the mucous membranes. We expect in this author's next volume the full and explicit information a fact of such import calls for. The statement is not a novel one, however, for Dr. Blackall, in his work on Dropsies, has actually related cases of scorbutus and petechiæ, in which the urine was coagulable; but as the condition of the kidneys was not made a subject of enquiry, his observations, as well as many of the same stamp by other writers, must, at the present day, be acknowledged to be imperfect.

Our notes of interrogation show that we regard as exceedingly doubtful the possible admixture of albumen with the urine as a consequence of the absorption of pus or albuminous secretions. However, the notion that purulent depositions may be carried off through the kidneys has been held by a variety of surgeons, among the rest by Ambrose Paré, Desault, &c.; and recently M. Solon (p. 404) has availed himself of the doctrine, as one respecting the soundness of which no very serious doubts are to be entertained. And with respect to the passage of albuminous secretions, formed elsewhere, into the urine, we find Cotunnius,* in 1764, endeavouring to explain thereby the presence of albumen in the urine of a dropsical patient, whose anasarca rapidly diminished under the use of diuretics. But our incredulity is justified by the testimony of M. Rayer, who has for the last seven years in vain sought for pus in the urine of individuals, in whom the absorption of purulent depositions of various kinds was effected under his own immediate observation. It may not be correct, however, to deny absolutely the possibility of the excretion of pus under these circumstances, for in two cases of metastatic abscess of the lungs, liver, and kidneys, the tubuli and mamillæ of the latter organs were found gorged with a puriform fluid: these facts would be conclusive, were it not possible that the pus was produced by

* De Ischiade Nervosâ Comment. Viennæ, 1770, p. 30.

direct inflammation of the renal tissues. The doctrine of purulent absorption is unfortunately in every respect unsettled.

Some remarkable cases observed in India have been recently related by Dr. J. Mouat, (*Calcutta Quarterly Med. Journal*, July, 1837—see *Br. and For. Med. Rev.*, Vol. VI. p. 251,) as demonstrating that the excretion of purulent deposits from the liver may be accomplished through the kidneys, intestines, and lungs, independently of any direct communication between the hepatic abscess and those organs. We have attentively perused these cases, but without being convinced of the reality of the fact they are adduced to prove. Those in which examination after death took place, are alone, as is manifest, entitled to serious consideration; and here, admitting the urinary deposit to have been truly purulent, as it probably was, though the fact is far from proved, the post-mortem details are materially deficient; the condition of the kidneys, and especially of the urinary passages is most imperfectly described, and the author appears to have mainly trusted for his particulars to the reports of others. His opinions curiously exemplify the vacillation of medical doctrines: by the multitude all manner of mischief is ascribed to the passage of a drop, aye, even of a drop of pus in substance into the circulation, and patients pronounced to be all but lost, in whom the least symptom of such an occurrence is discoverable; on the contrary, Dr. J. Mouat attributes numerous recoveries to the passage of that fluid through the blood and kidneys into the urine, and the preservation of one man's life for eleven months, to his constantly relieving his liver of quantities of pus by this most extraordinary route. Bengal practice will no doubt supply Dr. Mouat with the opportunity of substantiating his assertions, if they be really correct, in such manner as to place them beyond the reach of critical cavils.

The fact noted by Cotunnius proves nothing, for the absence of albumen from the urine was not ascertained before the absorption of the anasarcous fluid; in similar cases, where this precaution was taken by M. Rayer, none of that principle was ever detected in urine, voided concomitantly with the disappearance of dropsical effusion.

B. *a.* In our second class we have placed essential hæmaturia, a disorder which of course causes the excretion of albumen along with or without the other principles of the blood. The presence of the coagulable ingredient in diabetic urine was first ascertained by Cotunnius (Op. cit. p. 31); no doubt of its frequent occurrence therein, though its amount and degree of persistence are by no means known, is expressed; nor is any hint, respecting its dependence on peculiar disease of the kidneys, let fall even by the most zealous followers of Dr. Bright.* According to Thénard, Dupuytren, and Dr. Graves, its appearance is an indication of approaching recovery; M. Rayer has, on the contrary, satisfied himself that when the urine becomes thus impregnated, the immediate occurrence of dropsy is to be apprehended.

It is a well known fact, that the urine of healthy individuals may become albuminous for a short while, for instance, twenty-four hours after direct or indirect excitement of the urinary passages. We are not quite sure of being right in ascribing the action of certain articles of food and medicinal agents to such intermediate irritation of the kidneys;

* See, however, Cyclopædia of Practical Medicine, vol. i., p. 543.

it is perhaps equally tenable that an altered status of the blood is, in these cases, the direct cause of the excretion of albumen with the urine. Further observations are necessary to allow of a decisive opinion on this point; meanwhile let us state the facts. Dr. Christison "has occasionally known a temporary albuminous impregnation, produced in healthy individuals by eating freely cheese, pastry, and such other indigestible articles as are known to have in general the effect of increasing the usual solid ingredients of the urine, and occasioning a large deposit of lithic acid and lithate of ammonia." (p. 36.) M. Solon contends that where such consequences follow, from the cause stated, individual predisposition must be admitted in order to account for them;—this is very possibly the truth.

Again, Dr. Christison has repeatedly seen the same condition of the urine, induced for a time (he does not specify what time) by a cantharides blister, when it excited symptoms of renal irritation.* In the only original case related by M. Rayer, as exemplifying the production of nephritis by the action of a blister, no albuminuria existed.

Dr. Mateer† has given us the history of a case of dropsy with incoagulable urine, wherein that fluid became distinctly coagulable during the use of infusion of gentian and carbonate of ammonia, the coagulability increasing on the addition of minute quantities of tinctura lyttæ to the medicine. The curative properties of these remedies seemed to be closely connected with the production of albuminuria. As this writer reminds us, Cullen had remarked that blisters "necessarily cause a great quantity of serum to be carried off by the kidneys;" and he, himself, further imagines that all stimulant diuretics cause an albuminous state of the urine, while they carry off dropsical deposits. We find no facts detailed confirmative of this hypothesis, which originated, as we have shown, with Cotugno, and has been proved fallacious by M. Rayer.

We find Dr. Blackall, again, recording his suspicion that the excessive use of another class of medicines, the alkalies and their salts, "disposes the serum of the blood to pass off by the kidneys."‡ There is some probability that such is really the case. It appears, indeed, to be generally recognized, not as the result of M. Magendie's speculations, but as that of the experience of such men as Huxham, Dr. Copland, &c., that those medicines, when used in excess, materially attenuate the blood in the end; now, if so, the condition of that fluid is artificially rendered the same as in scorbutic subjects, in whom the occurrence of albuminuria seems unquestionable. All this, however, like almost every other point connected with that phenomenon, requires deliberate investigation.

The testimony of writers respecting the action of mercury in this way is contradictory. The evidence of Dr. Wells, on the affirmative side of the question, must be allowed to carry considerable weight with it: this enlightened observer examined the urine of six syphilitic patients before they began to use mercury, and found that it contained no serum in five, and not more in the sixth, than "the smallest quantity that can be detected by heat or nitrous acid." After they had been in a state of salivation upwards of a fortnight, he reexamined their urine, and discovered

* Cruickshank states (Rollo on Diabetes, 2d Ed. p. 448,) that in strangury from cantharides, the urine assumes the appearance of a mass of hydatids.

† Edinb. Med. and Surg. Journ., Jan. 1837, p. 79.

‡ On Dropsies, p. 81.

that that which originally contained a little, now contained more; that in three other cases the foreign principle was manifestly present; while the remaining two individuals were completely free from albuminuria. Dr. Christison, too, has met with several facts "tending to prove the connexion of such a state with mercurial erythism;" a vague statement of this sort, however, is evidently valueless. On the other hand, M. Solon was unable to detect albuminous impregnation in subjects labouring under ptyalism, consequent on mercurial frictions, employed in a variety of diseases; and M. Rayer is of opinion that the urine does not become thus affected during salivation, unless scurvy, purpura, or an intercurrent affection of the urinary apparatus coexist therewith.

It appears from all this that the influence of mercury is far from being firmly established.

M. Solon's volume contains some cases seeming to show that hyperæmia of the kidneys, produced by common causes, will produce albuminuria; but it must be confessed that as no post-mortem examination was made in these cases, the exact condition of the organs is a matter of doubt.

*b.* We for the present pass over this division of our second class; the proofs of their being entitled to hold the place we have given them will be adduced when we speak of the different organic diseases enumerated.

*c.* The operation of this class of causes of albuminous admixture is easily intelligible.

The urine becomes loaded to a greater or less extent with semen, under a variety of circumstances. Thus it is well known that the secretion of the testes escapes into the urethra in certain cases of paralysis, and in consequence of excessive venereal indulgence, and that when the bladder is evacuated shortly after coition, its contents carry with them a certain quantity of seminal fluid. Again, a reflux of this fluid may take place into the bladder, immediately after its entry into the urethra, during the act of copulation, in cases of stricture of that canal; and M. Rayer has lately attended two persons, labouring under obstinate constipation, who voided pure semen while at stool; that the fluid was seminal, and not prostatic, was proved by the discovery of a multitude of spermatic animalcules.

The presence of these Entozoa is, according to M. Rayer, the only infallible sign of that of semen; for the nebulæ and sediments of spermatic urine are not, by their simple appearance, distinguishable from those produced by impregnation with mucus. The specific gravity of the animalcules being greater than that of the fluid in which they are suspended, they sink to the bottom of the vessel in twenty-four hours or so, and may then be easily recognized with the microscope. They may be detected in the sediment weeks after its deposition, for though urine kills them almost instantaneously, it retards their putrefaction. The absence of Spermatozoa does not, however, in all cases positively prove that the urine contains no semen, for in phthisical and many aged subjects the latter secretion contains but a very small share of animalcules; such at least is M. Rayer's statement. In cases where the urine is rendered turbid by imperfect semen of this kind, we may be guided in ascribing the muddiness to its true cause, as well by the absence of the microscopical characters of mucus, &c., as by these positive signs: acidity, *general* turbidity, which persists, but does not increase under the action of heat and nitric acid, and exhalation of the peculiar odour of semen during evaporation. We have been thus particular in enumerating the characters of seminal impregnation, because we are persuaded that its discovery may now and then become a matter of real practical importance. Dr. Willis somewhere remarks, in the work recently reviewed in this Journal, that such detection may occasionally give a clue to the origin of emaciation and cachectic symptoms, in cases where the practitioner had puzzled himself in vain in searching for organic lesions to explain the condition of his patient.

M. Rayer is acquainted with no method of distinguishing the secretion of the prostate gland when mixed with urine.

D. Cotunnius maintained that "all the vapours exhaled in the various cavities of the body as well as the urine become coagulable during disease." (*Morbi vi.*)* Our countryman, Cruickshank, believed that albuminous urine attended "every case of increased action of vessels, more particularly of the inflammatory kind."† Nysten announced in 1811 the presence of a large quantity of albumen in the urine of a subject who died rapidly of acute peritonitis, and appears to have conceived albuminous impregnation characteristic of inflammatory urine generally, though he allows it may sometimes depend on a particular condition of the urinary organs.‡ M. Rayer, also, to pass over a variety of evidence to the same effect, supplied by intermediate observers, states positively that the urine may contain albumen in acute affections for a period of several days, that its appearance almost always coexists with an increase of specific gravity, and of the healthy proportion of lithic acid and lithate of ammonia, and ordinarily indicates congestion of the kidneys, ureters, and bladder. M. Andral, too, declares that he has occasionally met with coagulable urine in the course of acute diseases.

But the most striking observations, appertaining to this matter, have recently been made public by M. Solon: to this gentleman the profession is beholden for the anouncement of a presumed connexion between the appearance of albumen in the urine and the establishment of a favorable crisis in acute febrile, and inflammatory disorders. We shall first lay an abstract of his facts before the reader, and then make a few remarks as to the claim of such urine to be termed a "critical sign." Upwards of one hundred pages of M. Solon's volume are occupied with a disquisition on this subject.

His cases are divisible into two distinct groups: in one of these the urine appears to have become coagulable by heat and nitric acid, precisely in the same manner as in Bright's disease; in the other, precipitable by nitric acid but not coagulable by heat; ebullition, on the contrary, here redissolves the precipitate occasioned by the acid. There can be no doubt that in the first group of cases the coagulation depended on the presence of albumen; and we are told that the foreign principle disappeared gradually with the advancement of convalescence: the action of heat showed that the precipitate in the second was not of albuminous nature, and the microscope in the hands of M. Donné, that it was really

* Op. cit., p. 31. † Rollo on Diabetes, p. 444.
‡ Recherches de Chim. et de Phys. pathologiques. Paris, 1811, p. 253.

composed of lithate of ammonia. The following table gives at one view the results of M. Solon's experience on this matter:

| Diseases. | | Number of Cases. | Coagulability: Not produced by any reagent. A — Recovered | Died. | Produced by heat. B. — Recovered | Died. | Produced by nitric acid. C. — Recovered | Died. |
|---|---|---|---|---|---|---|---|---|
| Intermittent fevers | | 8 | 1 | 0 | 2 | 0 | 5 | 0 |
| Febrile Exanthemata. | Measles | 7 | 2 | 0 | 1 | 0 | 4 | 0 |
| | Variola | 11 | 6 | 0 | 1 | 0 | 4 | 0 |
| | Scarlatina | 3 | 1 | 0 | 1 | 0 | 1 | 0 |
| | Pemphigus | 1 | 0 | 0 | 0 | 0 | 1 | 0 |
| Typhoid fevers | | 23 | 3 | 1 | 3 | 1 | 15 | 0 |
| Bronchitis | | 1 | 0 | 0 | 0 | 0 | 1 | 0 |
| Pleuropneumonia | | 24 | 2 | 0 | 2 | 0 | 17 | 3 |
| | | 78 | 15 | 1 | 10 | 1 | 48 | 3 |

Hence it appears that the urine of $\frac{1}{11}$ of a mass of patients affected with acute diseases may be expected to become albuminous at some period or other before their recovery. The cause of this phenomenon is not easily demonstrable; M. Solon himself seems inclined to ascribe it to "a modification of secretion, occasioned by a nervous influence;" an hypothesis which cannot be said to boast of much plausibility. But no matter how they are to be accounted for, it is manifest that these facts, if correctly observed, clash with the too prevalent notion that albuminuria is pathognomonic of a certain affection of the kidneys; it is right, however, that the reader should be put in possession of Dr. Christison's mode of attempting to evade their force. We quote his words: "It is fair and reasonable to throw out the query, whether, considering that urine decidedly albuminous is a very rare concomitant of the crisis in these acute diseases, the cases where it does occur may not actually be instances of a tendency to granular deposition, arrested along with the local and general reaction which it accompanies? The affirmative is a perfectly reasonable answer. Singularly enough, in the only two cases where I have observed coagulable urine during the crisis of acute diseases, namely, in two cases of acute pneumonia, the progress of matters unequivocally proved that the patients laboured under granular degeneration of the kidneys." (p. 42.) In this view, however, Dr. Christison has been anticipated, strangely enough, by M. Solon himself, who has tendered it in explanation of Dr. Gregory's case of typhoid fever with coagulable urine, while he has totally overlooked its applicability to his own cases. It was certainly incumbent on Dr. Christison to publish accurate histories of the cases to which he alludes, and so allow the medical world to judge for itself whether their progress was really such as he presumes it to have been. The question is too important a one to be decided by mere assertions.

The second group of cases is unimportant in respect of the presence of albumen; but, combined with the first, it shows that *precipitable* urine may be looked for in very nearly three fourths of all cases of acute febrile disease. It remains to be asked whether these conditions of the urine really constitute a "critical sign" of the resolution of the affections during which they are observed? By referring to the Table (p. 133), it will be seen to contain nineteen exceptional cases in this point of view: 15 in the columns A; 1 in those headed B; 3 in those marked C. M. Solon must be heard in explanation of these exceptions: In respect of the 15 cases of recovery wherein no precipitate occurred, he reminds us that the kidneys are not the sole critical emunctory,—that the skin and intestinal canal should not be forgotten; and that the greatest number of exceptional cases were of smallpox, where the skin may be supposed "to be specially charged with the critical movement." Again, the patient who died in spite of his urine having become albuminous, "was affected with splenitis, and *may have* suffered from absorption of pus, which, in its turn, *might* have caused the passage of albumen into the urine." We have already adverted to the probability of purulent urine being thus induced. Lastly, the phenomenon existed "in a very moderate degree" in the exceptional pneumonic cases. The reader will form his own judgment of the validity of this author's defence; he will probably be guided therein by learning that coagulability occurred in "a transient and, *as it were*, insignificant manner at various periods of the diseases mentioned" (here M. Solon confirms the statements of previous writers); "frequently supervened, and then disappeared, *because* other morbid phenomena declared themselves; but finally appeared again, to announce definitive resolution."

It is not a little remarkable that Cruickshank (Op. cit., p. 448,) believed the *disappearance* of albumen from the urine to point out the same favorable turn in the course of inflammatory affections, which is indicated, according to the French writer, by its *appearance* therein. A third version, possibly, however, coinciding with that of Cruickshank, comes from Dr. Mateer (Loc. cit., p. 92); he conceives that albuminuria occurs towards "the turn of fever; that after the kidneys have for some time secreted albumen, this suddenly disappears, and lithic acid and the lithates are formed in its place, and in this way the *crisis per urinas* is effected." The truth is that albuminuria is an occasional concomitant of acute diseases; but what relation it may bear to what is termed their crisis is utterly unknown; how, indeed, could the fact be otherwise, when the reality of that crisis is still a problem?

Dr. Copland states that he has observed coagulable urine frequently in the acute diseases of children, "where no alteration of the kidneys existed." These facts are unimportant, according to Dr. Osborne, because the urine is quite differently constituted in children and adults. We know not whence Dr. Osborne has derived his information, which is wholly irreconcilable with the results obtained by modern chemists of repute.

The belief in the occasional presence of albumen in the urine of hysterical subjects seems to rest on an analysis made by Peschier in 1826.* It is impossible, however, even to guess what may have been the organic condition of the individual supplying the urine examined; indeed, the title given to the article is the sole evidence of the existence of hysteria; and its author does not appear to have been a medical prac-

* Journal de Chim. Médicale, vol. ii. p. 234.

titioner. Both Nysten and M. Solon have in vain sought for a particle of albumen in the urine in various nervous affections.

When Dr. Copland, in imitation of Dr. Wells, speaks of albuminous flocculi being deposited during the continuance of the anasarca, occasionally succeeding scarlatina, he probably confounds mucous nebulæ with the coagulable principle; but that the latter is discoverable under those circumstances frequently, if not constantly, no practical observer now disputes. The condition of the kidneys in these cases is, however, not yet decisively settled. Burserius, Fischer, Dr. Blackall, and others have furnished evidence of their presenting more or less marked signs of inflammatory action; but accounts of dissections published before the general promulgation of Dr. Bright's doctrine, need not, for obvious reasons, be examined at any length. Thus limited as to the date of our documents, the earliest communication we find on the subject is that of Mr. Hamilton.* In several cases of death occurring in patients anasarcous after scarlatina, that observer found the kidneys diseased; and, on showing accurate drawings of their lesions to some Edinburgh professors, these gentlemen pronounced them to represent specimens of the early stage of Bright's disease; the descriptions given in the essay justify their decision. Cases are also related here, showing that the urine may be coagulable and the kidneys diseased, before the termination of the primary fever.

MM. Sebastian and Constant and Dr. Mateer give their testimony to the correct observation of Mr. Hamilton; in the volume before us, too, Dr. Christison supplies two cases (x. and xxvi.) which he regards as unequivocal examples of Bright's disease, produced by the renal irritation consequent on scarlatina.

On the other hand Dr. Graves† has, as he avers, seen a case of dropsy after the eruption in question with highly albuminous urine, wherein the kidneys "presented a perfectly healthy structure;" and Dr. Willis (Op. cit., p. 157,) is led to conclude that structural disease of those organs could not have been the cause of the albuminuria almost constantly observed by him to accompany the condition in question. We regret sincerely these authors should have deceived themselves into the belief that vague impressions—and these, in the instance of the latter, almost dubitatively stated—could have any weight in the decision of a question of facts like the present. Had they faithfully recorded the cases to which they refer, they would have done good service to renal pathology; as it is, they have simply excited doubts, without producing a particle of conviction. Unfortunately their error is common to the majority of the medical writers of the day. Open any work you please, and with the rarest exceptions, you will search its pages in vain for well-observed *facts*, and as surely will your equanimity be ruffled by the constant recurrence of egotistical displays of empty *opinions*.

We are not aware whether any investigations have been made into the condition of the urine in the anasarca following, in some instances, the other exanthemata.

It has been asserted that the urine is sometimes albuminous in gout;

* Edin. Med. and Surg. Journ., Jan. 1833.

† Med. Gazette, Oct. 20, 1838.

no particulars of the organic condition of individuals thus affected are given by the authors of the assertion.

Organic diseases, uncomplicated with renal lesion, undoubtedly form the most important class of cases, distinct from those of Bright's disease, wherein albuminuria may occur,—inasmuch as the existence of that symptom is in them most likely to betray the observer into an erroneous diagnosis. Dr. Darwall was the first to bring forward an example of the coexistence of albuminuria, dropsy, disease of the heart, and "perfect soundness" of the kidneys.* Professor Forget's seventh and eighth cases exemplify a similar combination of circumstances,† and M. Solon has added another to the list. Dr. Christison, however, strives to deprive the facts of the French observers of their importance, by hinting that they were not perfectly competent judges of the morbid anatomy of the kidney, at the time they met with them. On this we have to observe, that M. Solon is to our knowledge a man of most sound general information, and had, besides, been at least a year engaged in the study of renal pathology at the period in question. And, as respects M. Forget, the attestation of the absence of renal lesion is clear and straightforward; and further, given unusual weight by the fact that its author actually expresses vexation at his inability to find a trace of disease in the kidneys. We must warn the reader, too, that it is utterly incorrect to state, as Dr. Christison does, that M. Forget "appears always to have looked for some degree of granular deposition;" on the contrary, he pointedly declares that he adopts the term albuminuria, because such deposition is not a constant phenomenon.

M. Solon narrates the history of a subject who died with anasarca, bronchitis, diseased intestine, and healthy kidneys, and had, during life, discharged albuminous urine. Dr. Morrison, again, has recently given the particulars of a case of aneurism of the ventral aorta, which terminated fatally by rupture and effusion of "at least four quarts" of blood into the abdomen; the kidneys were found in every respect natural, except "from their seeming, like the other abdominal viscera, totally devoid of blood;" yet during the previous *five years* the urine had at various periods been found coagulable by heat and acids. The medical attendant's persuasion, shared in by all who had seen the case, that his patient laboured under special renal disease seems a fair guarantee of his having examined the kidneys with all necessary care.‡

M. A. Toulmouche has swelled the list of such facts by the relation (*Gaz. Médicale*, Fev. 1839,) of a case of phthisis, attended with chronic peritonitis and ulcerations in the intestines, wherein the urine furnished a thick stratum of albumen, when treated in the usual manner, though on the death of the patient the kidneys were found "of small size, pale, and perfectly healthy."

Much has been written on the connexion between dropsies and albuminous urine, especially with a view to proving the latter a dependence on the former. Attempts have even been made by Cruickshank and Dr. Blackall to show that albuminuria exists only in the form of dropsy unconnected with organic disease, and hence to establish a method of

* Cyc. Prac. Med.—Art. "Dropsy."

† Gaz. Méd. vol. v. p. 609.

‡ Dublin Med. Journal, No. xxxvi., 1838.

diagnosticating the two kinds of serous effusion. It is needless to dwell on the fallacy of this view, the converse of which would be nearer the truth. That coexisting albuminous impregnation of the urine and dropsical effusion stand to each other in the relation of effect and cause may be doubted, for the following reasons: 1. Coagulable urine is voided in cases where no dropsy exists. 2. When dropsy disappears under the influence of diuretics, the urine does not therefore become coagulable. 3. No cases of dropsy occurring in subjects proved to be free from organic disease or a morbid condition of the blood, and in which the urine has been found albuminous, are on record.

Such seems to be a tolerably accurate enumeration of the various conditions under which albuminuria has been observed. From the facts related or referred to, the subjoined propositions immediately derive:

1. To infer the existence of a special lesion of the kidneys from the mere presence of albuminuria is utterly incorrect.

2. Consequently, boiling the urine of all the inmates of an hospital, according to the plan of certain observers, in order to determine the frequency of Bright's disease, is liable to lead to false deductions.

3. It will be necessary for future observers to specify the condition of their patients in respect of all the agencies presumed to give rise to a discharge of albumen with the urine, in order to entitle them to refer its appearance, indisputably to any one of those agencies rather than another.

4. The characters of the albuminous precipitate itself, as well as those of the urine containing it, must be much more carefully noted than has hitherto been habitually done.

M. Rayer terminates a very lucid enquiry into the authenticity of cases, reported as examples of the discharge of milky urine, by a number of propositions which we condense as follows: The existence of *naturally milky* urine, at present rests on the mere testimony of authors, never having been proved by rigorous experiment; the only legitimate inference, from cases hitherto published, is that the presence of an abundance of fatty matter in the urine will give that fluid a lactiform appearance: casein and the true milk globule, the only incontestable evidences of the presence of milk, have never yet been recognized in urine, unless in cases where the two fluids had been artificially mixed. Dr. Aldridge's case, as reported by Dr. Graves, and faithfully translated in the pages before us, is inconclusive, according to M. Rayer, of the fact it is adduced to prove; because, though Dr. Aldridge states his having detected casein, he leaves us in the dark as to the method he employed in his search for it, and does not describe its characters with accuracy. Dr. Graves will, we doubt not, see the reasonableness of M. Rayer's demurrer, and probably induce his friend, provided accurate note was made of them at the time of their performance, to give his experiments publicity.

M. Rayer admits that the materials of the bile pass into the urine in various affections of the liver, and in all diseases attended with mechanical obstruction to the flow of the hepatic secretion; to these M. Solon adds the "occurrence of the disordered state of secretion, known by the ancients as the *bilious state*." Nearly forty pages of the latter gentleman's book are occupied with an enquiry into this subject, whereon he appears to fancy himself much more of an original observer than he really is. The whole doctrine was distinctly stated by our most sagacious countryman, Dr. Cruickshank, in his additions to Dr. Rollo's work: "In jaundice," he says, "the state of the disease may be ascertained by examining the urine, in which a very small quantity of bile may be detected by the nitric or muriatic acids," &c. &c.

M. Rayer has recognized the peculiar action of ammonia on pus, whereby it converts that fluid into a ropy, slimy matter, varying in consistence with the quantity of alkali present. He is induced to believe that the ropy matter, detected in certain specimens of urine, such as those voided in chronic cystitis, &c. is not secreted in that state; an opinion which rests on his having repeatedly followed the conversion of pus in acid urine into a ropy, "catarrhal" matter, in proportion as an alkaline reaction was established by the development of ammonia. Our readers will here discover the conformation of Dr. Babington's ingenious researches in the same direction: their accuracy is further attested by Dr. Bright, who "has at this time under his care, a lady, labouring under copious purulent discharge from the kidney, which, on one or two occasions, has been passed in the form of mucus, owing to the administration of alkaline remedies."

If a drop of acid urine, rendered turbid by impregnation with pus, be submitted to microscopic inspection, immediately after emission, and before the foreign fluid has had time to gravitate to the bottom, a vast number of globules are detected. These are regular and spheroidal, measure about one hundredth of a millimeter,* have a well-defined border, a semi-transparent white surface, and a granular appearance, owing to the presence of a number of minute grayish granules in their substance. The changes these globules undergo, by the progress of putrefaction, are well described; but we regret to find the announcement, already made by M. Donné, confirmed by M. Rayer, to the effect that no distinctive character between these and mucous globules is detected with the microscope. If not by their globule, these fluids are, according to our author, otherwise distinguishable; pus or muco-pus contains albumen and fatty matter, mucus does not. The presence or absence of pus or muco-pus in a fluid of doubtful character may be, therefore, easily ascertained by the action of nitric acid, heat, and ether.

From the very comprehensive details that follow on the passage of medicinal agents into the urine, on the nebulæ, sediments, and cremors of that fluid, and on the phenomena of its putrefaction, we have room but for one or two extracts. It appears, as we have already mentioned, from the researches of M. Quevenne, that a formation of *blackish globules* takes place in acid urine, presenting an amorphous sediment shortly after emission, from the third to the twentieth day after that process. They are composed of superlithates, ordinarily of ammonia, measure one hundredth of a millimeter in diameter, and are frequently mixed with crystals of neutral ammoniacal phosphate of magnesia. When urine of this description grows alkaline, a new species of globules makes its appearance: these are white and *organic*, of new formation, and measure only $\frac{1}{1200}$ of a millimeter in diameter; the lithates very rarely assume the globular

* The millimeter is equal 0·039370 of an English inch.

form in alkaline urine. A third sort of globule has been detected in the sediment of diabetic urine by the same experimenter; this, from his description, appears to be identical with the ordinary *yeast-globule.*

In his chapter on the relations presumed to exist between certain morbid conditions of the urine and those of the blood, and other fluids, M. Rayer has given a luminous statement of the whole fund of knowledge as yet accumulated on this most important topic: we shall in due season avail ourselves of its most practical matter.

In examining the body of our author's work, our attention is first arrested by the section on mechanical lesions of the renal organs, under which head are comprehended *wounds, concussions, contusions,* and *lacerations.* The general impression derived from an attentive study of the facts, narrated in this division of the book, is the exceeding difficulty of forming an estimate, immediately on the occurrence of the accident, of the probable results of the injury sustained by the kidneys; cases have gone on in the most favorable manner for weeks even, and at the end of that time terminated fatally in a few days by the renewal of internal hemorrhage. The practitioner acquainted with the nature of previous experience in such cases will, therefore, be cautious in his prognosis to a degree that may appear extravagant to an ill-informed man. On the other hand, recovery has occasionally occurred where the most sanguine might well have despaired; of this abundant proof is given by the remarkable case, published by our countryman, Hennen, wherein complete restoration to health followed the discharge of a piece of cloth by the urethra, eight months after a gun-shot wound in the loins. M. Rayer, indeed, seems of opinion that the severity of these cases does not so much depend on the lesion of the kidney, *per se*, (except when the pelvis, calices, or renal vessels are wounded,) as on the accompanying injury done to neighbouring parts, such as the intestine, peritoneum, &c. This position is founded on a fallacious application of the results of experiments on the lower animals to the human subject:* the cases related in his pages prove it to be untenable, though we admit they show that, with the proviso stated, these injuries are on the whole less dangerous, perhaps, than is currently believed. Those which prove most quickly fatal affect the renal vessels.

Here is a statement which if not new is yet sufficiently important to excuse repetition: "discharge of blood by the urethra, and pain in the

* As Professor Marjolin very justly observes, (Dict. de Méd., i. 158,) were we to judge from experiments on the lower animals, wounds of the peritoneum itself must be looked on as almost unattended with danger. But the truth is, that any absurdity may be quasi proved by a clever use of analogical argument. Voltaire in his inimitable tale *Micromégas,* makes one of the *dramatis personæ* assert that the planet Mars is provided with *two moons.* In anticipating arguments against so notoriously false an assertion, he refers the incredulous to those who reason from analogy, as to persons who will prove the thing as clearly as that two and two make four:—he but justly ridicules the pretensions of such sophists. What then shall we say of the cruelties of M. Magendie—of the pertinacious enthusiasm with which he strives to foist on us the results of his complicated tortures of dogs, pigeons, and guinea-pigs as invariably applicable to the phenomena of the human system? What too of the wretched vanity that instigates him to repeat in public over and over again, year after year, experiments which he himself assures us have, Heaven knows how long, been proved to be conclusive of anything and everything he likes? What of the humanizing influence such unjustifiable proceedings must exercise on the youths who witness them?

region of the kidney, following an injury in the loins, are not infallible signs of a wound of the viscus in question, *unless the hemorrhage follow the injury almost immediately.* Traumatic inflammation of the lumbar muscles and cellular membrane may excite such inflammation of the kidney as shall in its turn cause the pain and hæmaturia alluded to. The diagnosis will be clear, if urine, or a fluid impregnated therewith, escape by the external opening." (p. 249.) On the other hand, in cases of contusion at least, the cortical substance has been superficially wounded without a drop of blood being discharged by the urethra: this is easily comprehensible. Lacerations of this kind, indeed, appear to be quite as curable as simple ecchymoses; an effusion of blood or coagulable lymph between the edges of the solution of continuity prevents the extravasation of urine, and subsequently effects cicatrization. This is well illustrated in our author's first plate.

M. Rayer's experience is confirmative of a most interesting announcement respecting the etiology of calculus made some years past by Mr. Earle:* he has found that traumatic lesions of the kidney predispose to calculous affections, and especially to certain forms of gravel. The occasional recurrence of dull local pain in the lumbar region after such injuries, even though it appear only at distant intervals, and last on each occasion for an extremely short time, appears to indicate, with some certainty, this unfortunate tendency. The inference from this is that his medical attendant should never lose sight, if possible, of a patient who has suffered renal injury, nor fail to combat energetically the slightest symptoms which may be traced to the temporary manifestation of an habitually latent inflammation.

There is nothing novel in the mode of treatment recommended for these accidents; this is as it should be, inasmuch as M. Rayer seems to have had no personal experience of them in their early stages. We can, nevertheless, recommend this chapter as one to which, from the excellence of its arrangements and abundance of its borrowed facts, the practitioner may refer, on an emergency, with almost complete certainty of finding the information he requires.

Passing on to inflammatory diseases of the kidneys, our author commences by dividing these affections into three groups, according to the tissue they immediately occupy: each of these groups is subdivided into a certain number of species, distinguishable from each other, either by the cause producing them, or by some particular symptomatic character in the malady. Here is the classification.

| | |
|---|---|
| 1. NEPHRITIS. Inflammation of the cortical or tubular substances. | 1. *Simple.* 2. *Produced by morbid poisons.* 3. *Arthritic, i. e. Gouty and Rheumatismal.* 4. *Albuminous.* |
| 2. PYELITIS. Inflammation of the pelvis and calices. | 1. *Simple.* 2. *Blennorrhagic.* 3. *Calculous.* 4. *Verminous.* |
| 3. PERINEPHRITIS. Inflammation of the external cellular and fibrous membranes of the kidneys, or of their investing adipose cellular tissue. | |
| 4. PYELONEPHRITIS. A combination of Nos. 1 and 2. | |

* Med. Chir. Trans., xi. 211.

That the anatomical distinctions, assumed to exist between renal inflammations by this arrangement, are really to be found in nature, the subsequent pages even of the present volume very fully show; it is most gratifying to discover in this separation of nearly similar disorders, decided evidence of increased accuracy of knowledge of renal disease, and we are willing to wait patiently for the labours of future observers to point out how these different local states, for this M. Rayer has failed to do, are in all cases to be symptomatically distinguished. As to the four species of the first group, Nephritis, these, when fully developed, differ from each other in respect of their symptoms and anatomical characters, while they all agree in commencing by partial or general hyperæmia. Thus we are told, in respect of the organic changes induced by each, that deposition of pus is a frequent termination of the simple variety; that accumulation of plastic lymph or lithic acid, in the cortical substance or mammillæ, is very often remarked in the arthritic species; that gangrene more particularly characterizes the form of the disease produced by "infection;" and that the most ordinary appearances in the albuminous variety are anæmia, consecutive to hyperæmia, increase in size and weight of the affected organs, and milky spots or granulations. Induration and discoloration are common to all the species except the third. To the general correctness of this statement, no serious objection can, we believe, be offered; but as we proceed, it will be found that here, as elsewhere, nature transgresses the limits set her by artificial separations.

The remainder of the present volume is devoted to the history of the subdivision *simple nephritis* alone, illustrated by 104 reported cases, of which 53 are original, and 51 selected from various authors, of very questionable scientific reputation in the majority of instances. To such analysis of this vast chapter (it contains upwards of 300 pages), as our space will permit, we at once proceed.

Mechanical agencies hold a prominent place among the causes of nephritis, as admitted by M. Rayer: of these two distinct kinds may be enumerated, wounds and analogous injuries, and the irritation of foreign bodies, such as calculi, worms, or urine abnormally retained in its excretory passages, and more especially in the pelvis of the kidney. From this it follows that all morbid states, inducing such retention, are really indirect causes of renal inflammation; thus various affections of the ureters, prostate, urethra, and bladder, displacements and diseases of the uterus, ovaries, rectum, &c., nay, even cerebro-spinal maladies, through the paralysis they immediately, and the retention of urine they mediately occasion, rank among the causes of simple nephritis. And it further follows, that advanced age powerfully predisposes to the complaint, for all the morbid conditions of the genito-urinary organs, &c. that we have named are far most frequent at that period of life. Experience corroborates this inference; the occurrence of simple nephritis in children under seven years of age is exceedingly rare. In all these cases, inflammation of the pelvis precedes that of the substance of the kidneys. Cantharides, nitre, and similar agents, our author is of opinion, very rarely produce the disease: a notion carried still further by Professor Chomel, who doubts that a single unquestionable example of such causation is to be found:* this doctrine however, we are firmly convinced,

* Arch. Gén. de Méd., Jan. 1837.

and there are cases in our author's volume justifying this conviction, is by much too exclusive. Cold and damp, and the extension of a contiguous inflammation, terminate M. Rayer's catalogue of causes. On the influence of sex he has nothing of a positive kind to offer: the liability to the indirect causes of the complaint seems *à priori* to be pretty closely equal in the two sexes.

Influences which act at once on both kidneys usually excite inflammation in both simultaneously, but the disease may exist in a very different degree in the two organs. Again, when inflammation of one kidney is produced by mechanical agencies acting directly thereon, a temporary influence on the opposite organ is clearly manifested in the majority of cases by a material diminution or total suppression of the urinary secretion. Further, one organ only inflames in some instances, where the cause (e. g. the irritation of cantharides, cold and damp, &c.) should, to all appearance, act equally on both: in this fact we recognize a further illustration of the unfrequency of coexisting inflammation in symmetrical organs, which observation has established to be one of the general laws of inflammatory action.

We turned with very considerable anxiety to the symptomatology of acute nephritis; but we must confess that the results obtained by our author do not, by the new light they throw on its diagnosis, respond to the high estimate we had formed of their probable importance. Before the publication of this volume, it was fair to ascribe our imperfect acquaintance with the symptoms of nephritis to the fact that no observer had methodically and earnestly entered upon the investigation of this and cognate diseases; we are now constrained to admit that there must be some inherent difficulty attached to their study.

Respecting the rigors and febrile reaction of invasion, we are told nothing of much importance: the former are pronounced to occur in every case without exception, a statement which we are persuaded is incorrect. (Vide M. Chomel's paper.) The characters ascribed to the pain in nephritis are these: it may be superficial or deep seated; limited to a small or spread over a large surface; so intense as to be materially aggravated by the slightest touch, or so obscure that forcible alternate pressure on both kidneys is requisite to show which is affected; increased by movements of the trunk, and occasionally by decumbiture on the affected side and by the heat of the bed; remittent; rarely pulsatile (this is, on the contrary, an ordinary character of the pain in perinephritis,) and irradiating upwards to the diaphragm, or downwards to the bladder, groin, and testicle, or uterus and round ligament in the female. The bladder, strangely enough, is sometimes the principal if not the sole seat of the pain, in cases wherein that viscus is found to be in an almost perfectly healthy state after death, while the kidneys are seen to be profoundly disorganized; this peculiarity was long since noticed by Morgagni. Painful retraction of the testicle sometimes occurs, but is a more frequent attendant on pyelitis and calculous ureteritis.

M. Rayer's first plate includes an engraving of a kidney so enormously enlarged in the course of acute inflammation (it weighed 17 oz.), that a tumour was detected during life, both by application of the hand and by percussion; with similar cases, however, we must very rarely expect to meet. According to our observer's experience, the temperature of the superjacent skin is not increased, unless there be coexistent perinephritis or extra-renal abscess.

The modifications of the urine in this affection affect its quantity, visible qualities, and composition. The secretion is always diminished in quantity, and sometimes wholly suspended: (M. Rayer says of the latter "*especially* when both kidneys are affected;" we find no instance of such suppression, however, in his narrated cases, except where both organs were diseased.) The urine may be excreted rarely, twice or thrice in the twenty-four hours, or there may be distressing micturition. The chief changes in point of composition are stated to be caused by impregnation with blood or albumen, by diminished acidity, neutral reaction or alkalescence, and occasionally by admixture with pus. It is affirmed in the most distinct manner, that the discharge of sanguinolent or albuminous urine is not peculiar to the traumatic variety of nephritis, but that it may also occur when the attack proceeds from general causes or diseases of neighbouring parts. Its appearance is accidental and temporary, and may be completely put a stop to—unless when dependent in part on a coexisting affection of the bladder or urethra—by venesection. Now, in true albuminous nephritis (Bright's disease), the presence of albumen in the urine is a constant condition at all its periods; bloodletting will not cause it to disappear, and the quantity of the foreign principle, as well as of the urine voided, is always greater than in the simple variety of the disease: the coagulable excretions of both are hence sufficiently distinguishable.

These statements call for a remark or two. In the first place, the fact of coagulable urine being excreted in simple nephritis, is of such importance as to require numerous well-observed facts for its substantiation: here the onus probandi rested with M. Rayer, but unless assertions, of which he is no niggard, be taken for proofs, he has declined it almost completely; no case of acute nephritis is related in his volume wherein such a phenomena occurred, and three cases of the chronic form (in one of these the urine was purulent, the prostate and pelvis of the kidney diseased—in another there was cystitis and chronic enteritis,) are all that have been mustered in its exemplification. But while we reprehend this slovenly omission (for we doubt not M. Rayer has met with many more cases which he considers confirmative of the assertion he has made), we confess ourselves disposed to believe that assertion well founded. When the urine is mixed with pus, or tinged with blood, no doubt can be entertained on the matter; and M. Solon has reported at least three cases of acute and chronic simple nephritis attended with albuminuria: the ground on which this observer distinguishes the chronic case from Bright's disease —namely the absence of anasarca—is more debateable, however, than he seems to imagine. Secondly, there is an error in M. Rayer's declaration of the constancy of the presence of albumen in all the stages from first to last of Bright's disease; Dr. Bright himself and Dr. Christison have both noted its occasional total disappearance in that disorder. Thirdly, we dissent altogether from the correctness of taking the comparative abundance of urine discharged in Bright's disease as a ground of diagnosis between the two affections; in the acute stage of that disease, the secretion from the kidneys may be scanty, as well as in simple nephritis.

The urine of acute simple nephritis resembles that of Bright's affection by its diminished acidity and lowered proportion of lithic acid and lithates.

In the former disease, when chronic, alkalescence is more common and more persistent than in the latter; but the decrease in the proportion of the salts is least marked in the former.

The colour of the urine varies according as it contains blood, pus, mucus, &c.; it is usually pale when the disease is at its maximum (and not of the traumatic species), as it is then rarely impregnated with blood, and but slightly charged with lithic acid and colouring matter. M. Rayer makes an important rectification of the prevalent opinion that purulent urine is among the consequences of suppuration in the substance of the kidney: such condition of the fluid is on the contrary the ordinary effect of complication with inflammation of the mucous coat of the excretory passages. He has discovered depositions of pus in the cortical substance after death in cases wherein the absence of pus from the urine had been repeatedly established during life; and he denies that purulent impregnation ever depends on the presence of pus in the kidney, unless there be a direct communication between an abscess and the pelvis of the organ, or an ulcerative inflammation of the mammillæ.

Nothing is said of the density of the urine in the general description of the complaint; but, on referring to the table of specific gravities already adverted to, we find 1011·9 and 1015·6 noted as the mean result of five examinations in two subjects. The fluid was alkaline in these cases.

In the account of the general symptoms and local disorders affecting other organs in the course of the disease, we perceive nothing strikingly novel. Four forms of the malady, differing from each other in the general character of the symptoms, are enumerated; the *acute benignant*, the *inflammatory*, that in which *ischuria and cerebral symptoms* predominate, and a fourth remarkable by the tendency it manifests to *malignity and putrescence*.

M. Rayer prefaces his history of chronic nephritis by affirming that the symptoms of that affection have not yet been correctly laid down; pathologists having according to him fallen into a grievous error in ascribing the habitual excretion of purulent urine and the development of a tumour in the loins—which are in truth symptoms of chronic pyelitis—to chronic inflammation of the substance of the kidneys. If we are to trust his experience, "habitual pain in one or both renal regions, coexisting with diminished acidity, neutral reaction, or more especially alkalescence of the urine, and a sensation of weakness and numbness in the lower extremities" constitute the principal characters of chronic nephritis. But the confidence which this plain statement gives of encountering but little difficulty in the diagnosis of the affection is materially weakened by the subjoined qualification, that we are not justified even in suspecting the existence of the disease in many cases (*latent nephritis*) unless on the evidence of a most minute examination of the urine.

The major position M. Rayer seeks to establish respecting this disease, is that it exercises vast influence on the generation of phosphatic calculi. The urinary sediment in chronic nephritis, when the fluid itself is alkaline, is either amorphous and composed almost wholly of phosphate of lime, or chiefly constituted by crystals of ammoniacal phosphate of magnesia; or as most commonly happens, both these salts are held in suspension together with globules of mucus and a small quantity of lithates. The contents of the bladder are in ordinary cases excreted frequently and in small quantities at a time. Impregnation with blood or albumen is rare in the uncomplicated disease, with mucus frequent, with pus indicative of inflammation of the mucous membrane of the passages.

There is no fever, but the disease is attended with progressive emaciation, and a general cachectic state which, as M. Rayer declares, favours the development, among other morbid conditions, of pulmonary tubercularization. This may be true; but we must confess we shall wait for some more convincing proofs of a point so difficult of demonstration than a mere assertion, before we allow that it may not be false.

The diagnosis of this affection is occasionally confirmed by the excellent effects of local antiphlogistic measures; the renal pain, and the turbidity and alkalinity of the urine not unfrequently disappear altogether for a while after cupping in the loins.

It is plain that one of the most common and important varieties of what has been termed the "phosphatic diathesis" is portrayed in the preceding sketch. It has indeed long been known—and English writers were the first to advocate the notion—that injuries of the loins, and such diseases of the urinary passages as require instrumental treatment, induce the excretion of phosphatic salts with the urine. But these conditions are only its indirect cause,—the chronic nephritis, which they directly excite, has not, so far as we are aware, been yet put forward otherwise than vaguely and incidentally as a condition entailing an accumulation of phosphates in the urine.* On the other hand, it must be distinctly understood, as M. Rayer himself declares, that sensibility under pressure, which is the only symptom localizing the disease in the kidney, may be absent while all the other morbid phenomena of the phosphatic diathesis wear out the constitution of the patient: whether they are in such cases dependent on functional derangement of the kidneys only, or on latent chronic nephritis, can only be safely determined by post-mortem examination, and our author has as yet had no opportunity of appealing to its decision.

The anatomical characters of acute and chronic nephritis are next treated of, each receiving most beautiful and accurate illustration in the accompanying plates. We shall content ourselves for the present with enumerating them with all possible conciseness, as it will save repetition to reserve our more careful examination of them for another article. In the acute form, then, they are: 1. General or partial increase of size, according to the extent of the inflammation. 2. Various forms of injection, superficial and deep seated; visible with the naked eye, or requiring some slight magnifying power for discovery. 3. Red or anæmic induration of the two substances, coexisting with increasing weight and size; both states of coloration may be present in different parts of the same organ—a maculated appearance is the result. 4. Collections of pus, especially in the cortical substance; infiltration with the same fluid; ulceration of the

* A fact seeming to illustrate very clearly the influence of chronic nephritis on phosphatic urine is that lithic acid calculi (when they remain for any length of time in the passages) become incrusted with a layer of phosphatic salt, and this in spite of the constitutional tendency to lithic secretion. The irritation of the foreign body excites inflammation of the kidneys, and this in its turn the production of phosphatic urine.

mammillæ. 5. Gangrene (extremely rare). 6. Deposition of discoloured fibrine instead of pus (in the traumatic variety).

When the disease has reached the chronic stage, the following are its anatomical characteristics: 1. Diminished size of the kidney, if the affection be general; hypertrophy is, however, occasionally observed; increased weight and hardness of tissue. 2. Granulated, rough, or tuberculated exterior. 3. Red injection (possibly the result of an acute attack immediately before death). 4. Anæmia, partial or general, ordinarily attended with augmented density, and in many cases with hyperæmia; 5. A peculiar species of atrophy, of which one of the most remarkable characters consists in the appearance of cicatrices on the outer surface of the organ. 6. Ulceration of, or purulent collections in the mammillæ.

Their investing membranes frequently participate in the inflammation affecting the organs themselves—their vessels rarely.

We cannot tarry with M. Rayer's interesting remarks on diagnosis and prognosis, further than to state, that although, as we have already declared, his results on the former topic do not quite realize our expectations, the reader will derive solid instruction from their careful study. In the section on treatment, as was naturally to be apprehended, there is nothing remarkable for novelty in the general plan advised; but there is a sufficient share of practical importance in some of the particular details. Thus we are warned that in puerperal women nephritis has a marked tendency to terminate in suppuration; and that, notwithstanding the state of fatigue and weakness in which the function just performed may have left the patient, the lancet must be vigorously employed, more especially as the inflammation may have existed for some days when first recognized; the lumbar pain it induces may be readily mistaken for a natural consequence of labour.

M. Rayer believes that the best mode of preventing the occurrence of nephritis as a consequence of retention of urine produced by urinary or cerebral diseases, consists in evacuating the bladder carefully from time to time, and in *not* leaving a catheter constantly in the organ. We are glad to have an opportunity of recording so positive an opinion respecting the impropriety of employing the *sonde à demeure*, especially as it emanates from a practitioner of such repute as M. Rayer. When nephritis is induced by stricture of the urethra or prostatic disease, whereby the performance of catheterism is notably interfered with, leeches will be much more beneficially applied to the perinæum than to the loins.

Our author raises his voice against the exhibition of the mineral acids in cases of chronic nephritis with alkaline urine. We perfectly coincide with him in looking on their exhibition, whether as chemical reagents or as general tonics, as of most doubtful utility: they rarely succeed in altering with any persistency the constitution of the urine;* and the system at large almost invariably suffers from the gastric derangement they are so prone to induce. In these cases M. Rayer has directly and indirectly ascertained the excellent effects of nutritious and succulent diet, especially of the *animal* kind. Like all his predecessors, he recommends opium for mitigating the tortures of micturition; the drug may be

* Berzelius has published a very instructive case illustrative of their failure in this respect.

administered as an enema, a suppository, or by inunction, when its exhibition by the mouth is contraindicated: the frequent use of the emollient hip-bath is an excellent adjuvant to opiates.

The cases of acute and chronic nephritis related from the author's own experience, which occupy the remainder of the volume, constitute in our minds its essentially valuable part. They illustrate the obscure points in the history of the disease; they prove how erroneous is the opinion held by even some of the first pathologists of the day respecting its rarity; they exemplify the action of the various causes of the affection; mark out its relations to other disorders, and prove the modifications its symptoms undergo from the presence or absence of certain physiological conditions, such as pregnancy: each category of cases is enhanced in value by a luminous commentary.

Our article has already extended to such length, that we are unable to do more than thus state the nature of the information contained in the closing section of the work: this we can scarcely regret, however, for nothing but an intimate acquaintance therewith—very different from that obtainable even from a close analysis—will enable the student of renal pathology to estimate justly its important bearing on the practical history of the affection.

Of the fasciculi of magnificent plates accompanying the work, it is impossible to speak in terms too strongly encomiastic; both as delineations of morbid changes, and as works of art, they are truly admirable. To the volume we have reviewed are besides appended six engraved plates representing the microscopical appearances of various constituents of the urine, of albuminous lamellæ, of the globules of pus, mucus, and milk, &c.: these are really very convenient for reference, and illustrate excellently well the utility of this mode of studying organic products.

The appearance of M. Rayer's second volume will be the signal for our resuming the subject of renal diseases.

*Addendum.* Since these pages were sent to press, we have been very kindly favoured by M. Solon with a communication respecting some further researches which he has instituted on the subject of critical urine. Circumstances have as yet prevented him from publishing the particulars of these additional enquiries: we are now enabled to lay their result before our readers.

"The albuminous urine occasionally, and as it were, accidentally, observed during the progress of acute affections, is *not critical.* The presence of albumen in these cases depends either, as Drs. Gregory and Christison presumed, on a morbid state of the kidneys or bladder, on sanguineous exudation, or on a temporary modification of secretion. On the contrary, the urine which I have designated as simply *precipitable,* in contradistinction to that *coagulable by heat,* really announces by its appearance the solution of acute affections. It results from an examination of about 150 cases, that a critical precipitate is obtainable by nitric acid in seven out of every ten instances. When it presents itself, in the course of an acute disorder, in the form of a transverse disk of flocculent appearance, and four or five lines thick, the practitioner may rest satisfied of the approaching establishment of convalescence. This peculiar condition subsists one, two, or three days; subsequently the urine increases in density and usually becomes colourless; finally it gradually reassumes its normal characters."

Hence it appears that our unwillingness to abide by M. Solon's affirmation of the *critical* character of albuminuria, when occurring in acute diseases, has been fully justified by the event of further and unbiassed investigation. That it was unbiassed is proved by the result: the honorable candour evinced by M. Solon in thus unreservedly publishing his own refutation redounds infinitely to his credit.

Dr. Christison may have at one period held the opinion ascribed to him by M. Solon, but the observations on the subject in his present volume (p. 40), acquiescing as they do in the views originally adopted by the French writer, prove him to have since relinquished it.

---

